# Update in Spontaneous Coronary Artery Dissection

**DOI:** 10.3390/jcm7090228

**Published:** 2018-08-21

**Authors:** Joseph Ingrassia, Daniel Diver, Aseem Vashist

**Affiliations:** 1 Hoffman Heart and Vascular Institute, St. Francis Hospital and Medical Center, Hartford, CT 06105, USA; jingrassiamd@gmail.com (J.I.); ddiver@stfranciscare.org (D.D.); 2 Division of Cardiology, University of Connecticut School of Medicine, Farmington, CT 06030, USA; 3 VACT Healthcare System, West Haven, CT 06516, USA

**Keywords:** SCAD, cardiovascular disease in women

## Abstract

There has been increased awareness in the understanding and recognition of spontaneous coronary artery disease. Diagnosing this condition is of paramount importance as the treatment strategy differs greatly from traditional acute coronary syndrome patient. We review here the current state of management of spontaneous coronary artery disease.

## 1. Background, Definition and Pathophysiology

There has been an increased awareness and understanding of spontaneous coronary artery dissection (SCAD) in the last several years. From isolated case reports to prospectively followed cohorts, our understanding of the pathogenesis, natural history and optimal treatment options has increased significantly. Recently the American Heart Association and European Society of Cardiology have released documents aimed at guiding diagnosis, treatment, follow up and identifying gaps in knowledge [1,2].

SCAD is defined as a spontaneous dissection of the coronary artery that is not attributable to atherosclerosis, trauma, or instrumentation. Conventionally, coronary artery dissection refers to a tear in the intimal layer that produces a pathognomonic appearance on coronary angiography i.e., dye staining, multiple radiolucent lumens, visualization of an intimal flap, etc. In SCAD however, this appearance may only account for one quarter of the cases [3]. Contrary to the usual understanding of coronary dissection, SCAD can involve separation of the intima from the media with or without a disruption in the intimal layer and this can manifest angiographically as a subtle narrowing or caliber change of the vessel. The uninvolved distal vessel may therefore appear as a “plumped-up segment” This appearance is likely due to an intramural hematoma accumulating in the false lumen and resulting in the compression of the true lumen. This has been termed the medial hemorrhage hypothesis where the primary event is a disruption of the vasavasorum with resultant microhemorrhage in the media of the arterial wall [4]. SCAD may have intimal disruption though it is not clear if this is a primary event or secondary to extensive medial hemorrhage [2]. This unique pathophysiology of hemorrhage in the medial wall of the artery has significant treatment implications that differ from usual acute coronary syndrome patients. Given the strong association of SCAD with the female sex it suggests that there is a pathophysiologic role for female sex hormones that has yet to be fully elucidated.

## 2. Epidemiology

With more regular implementation of coronary angiography as well as intravascular imaging (intravascular ultrasound [IVUS] and optical coherence tomography [OCT]) SCAD is becoming an increasingly recognized entity. SCAD is overwhelmingly a disease affecting women with (up to 95% of SCAD patients are women). The true incidence of SCAD is difficult to ascertain owing to the limitations of coronary angiography in detecting SCAD and retrospective and historical databases that are likely to underestimate the true incidence. In a Japanese study that used optical coherence tomography imaging for 326 acute coronary syndrome patients, the prevalence of SCAD was 4% [5]. This however, may be an overestimation of the true incidence due to a high prevalence of men in the cohort and the possibility that some of the dissections were related to atherosclerosis. A population-based analysis of women presenting with acute coronary syndrome from the National Inpatient Sample from 2009 to 2014 showed that of the 752,352 patients undergoing coronary angiography, 7347 were diagnosed with SCAD, (approximately 1%) [6]. This likely represents an underestimation of the true incidence for the following reasons: (a) diagnoses were collected from ICD-9 discharge diagnostic codes, (b) awareness and recognition of different types of SCAD was not widely known at that time, (c) intracoronary imaging was underutilized, and (d) finally, only about 60% of female acute coronary syndrome (ACS) patients underwent coronary angiography. The true incidence is therefore, likely somewhere between 1–4% for all comers with ACS. The incidence in young and pregnant women may however be higher. The incidence of SCAD related ACS in young women under 50 years of age maybe as high as 24% according to a retrospective analysis of all coronary angiograms [7]. Similarly, according to a systematic literature review on pregnancy related ACS, up to 43% of cases were related to coronary dissection [8]. The mean age of SCAD patients is somewhere around 50 years old, though cases have been reported in women in their 80s [3]. These data offer a wide range of incidence plagued by the fact that the diagnosis may only be considered after the fact, or may be given in order to obscure periprocedural complications.

SCAD typically affects the mid to distal portion of the coronary arteries. Knowledge of the defining characteristics of the Saw Classification [9] (Table 1), and a high degree of suspicion can aid in making the correct diagnosis of SCAD in the right clinical context. Lack of atherosclerotic changes and extreme coronary tortuosity [10] in women with one or no coronary artery disease risk factors increases the likelihood of SCAD.

## 3. Risk Factors & Associated Conditions

SCAD has been strongly associated with fibromuscular dysplasia (FMD) with up to 86% of SCAD patients showing FMD in at least one noncoronary territory [11]. Despite the strong association of FMD with SCAD there have been only five cases of coronary FMD and SCAD reported [12,13,14,15,16]. This is likely related to low rates of autopsy and lack of widespread use of intracoronary imaging to diagnose coronary FMD. Recently Moulson et al. [13], described the histopathologic findings of coronary fibromuscular dysplasia in a patient that presented with an anterior ST elevation myocardial infarction secondary to SCAD who ultimately died of myocardial rupture. On autopsy, the patient’s right renal artery also demonstrated findings consistent with FMD [13]. Saw et al. have described marked intima-media thickening on OCT as suggestive of coronary FMD [17]. Perhaps with increased use of intravascular imaging, specifically OCT, a robust association of coronary FMD and SCAD will be established in the future.

**Figure 1:**
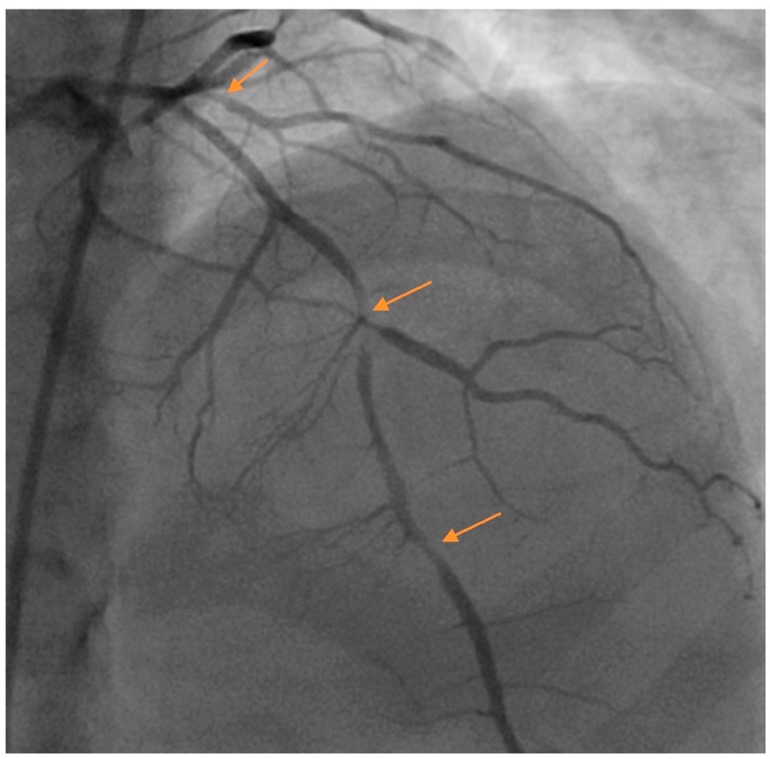

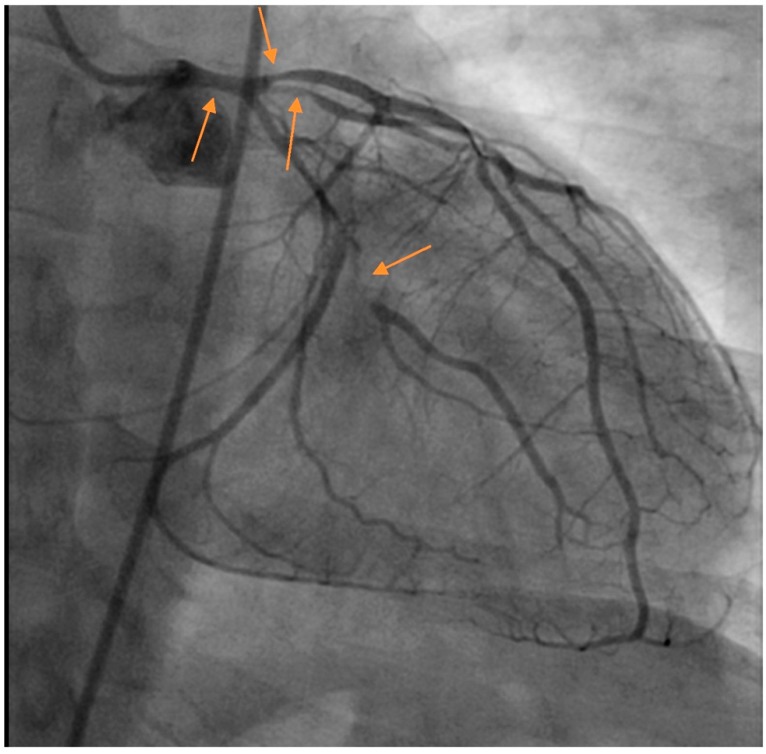
Multivessel spontaneous coronary artery dissection (SCAD) in a postpartum female presenting with acute coronary syndrome (ACS). Arrows denote areas of dissection. Images courtesy of Steven Cohen, M.D.

Pregnancy-related SCAD accounts for approximately 10% of SCAD cases [2], with most cases occurring in the immediate postpartum period [18]. The presentation is often comparatively more severe with a greater proportion of patients presenting with ST elevation myocardial infarction, ventricular fibrillation, proximal and multivessel SCAD [19] (Figure 1). Pregnancy related SCAD is associated with fewer extracoronary vascular abnormalities and possibly a lower risk of recurrence [18]. Estrogen and progesterone surges during pregnancy can cause medial degeneration of the arterial wall from decreased collagen synthesis and increased media mucopolysaccharide content, causing weakening of the vessel wall [20]. Whether or not other mechanisms are implicated is not well established. Patients suffering from pregnancy related SCAD are generally recommended to avoid future pregnancies. Table 2 lists several risk factors and suggested actions to take for patients with SCAD. 

## 4. Diagnosis

Despite the above-stated limitation of coronary angiography, it is still the recommended procedure to make the diagnosis of SCAD. Operators should have a high clinical index of suspicion for SCAD when evaluating patients with ACS especially women under the age of 60 or in the immediate postpartum period or women without the traditional risk factors for coronary artery disease. It is important to distinguish iatrogenic dissection versus SCAD and meticulous attention to details must be exercised in all patients during coronary angiography. Angiography in the SCAD population should be performed with exceedingly meticulous care as the rate of iatrogenic dissection in patients with SCAD can be as high as 3.4%, compared with <0.2% for all comers to the cardiac catheterization laboratory [21]. Additionally, forceful injections of contrast, risk propagation of the medial hemorrhage and/or intimal tear if it is present. Therefore, the diagnosis should be established with as few injections as possible.

**Figure 2:**
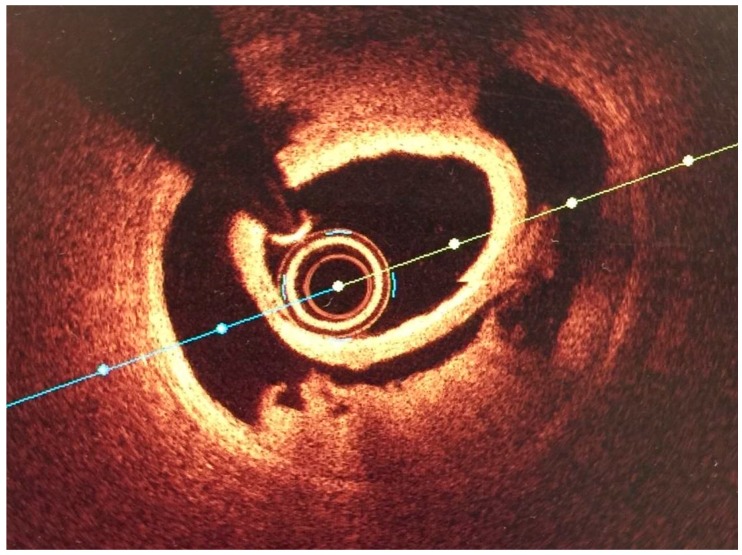
OCT Image of SCAD courtesy of Professor Mamas Mamas.

The Saw Classification [9] separates SCAD into three types. The first, type 1 is the classically understood definition of dissection with arterial wall staining and multiple lumens visualized on angiography. Type 2 SCAD angiographically appears as a long (>20 mm) diffuse smooth stenosis that often has a subtle and abrupt change in the arterial caliber. Type 2a demonstrates normal appearing arterial caliber proximal and distal to the vessel while in Type 2b the dissection extends to the angiographic vessel tip. Type 3 SCAD is described as a focal tubular stenosis that mimics atherosclerosis and requires a high degree of suspicion on the part of the operator and often intracoronary imaging (Figure 2). Extreme coronary tortuosity has been described as more frequent in SCAD patients than in controls [10].

Intracoronary imaging is a helpful to confirm the diagnosis of SCAD when the presentation is unclear such as in Type 3 SCAD. Intracoronary imaging can be helpful in guiding intervention (location and sizing of stents) in the event where intervention is necessary. Since most diagnoses of SCAD can be made angiographically and there is a risk of worsening the dissection by wiring the false lumen and/or passing an imaging catheter in fragile coronary arteries, intracoronary imaging should not be done solely to determine the extent of dissection. Briefly, OCT has greater spatial resolution but requires an injection of contrast which risks extending the dissection plane. IVUS has worse spatial resolution but better depth penetration and does not require an injection of contrast.

Coronary CTA is an attractive non-invasive option for making the diagnosis of SCAD but should be noted that dye penetration into the hematoma may not be present in Type 2 and Type 3 SCAD and that medial hemorrhage without intimal rupture may be a subtle, easily missed finding [22]. The spatial resolution of coronary CTA is decreased in the mid to distal vessels and may limit the specificity of coronary CTA as an initial diagnostic option. Coronary CTA may be a reasonable option in patients with known vessel dissection that have been managed conservatively to monitor healing. Roura et al. followed 20 SCAD patients managed conservatively with coronary CTA at 3–6 months post-dissection and found complete healing in 18/20 (90%) [23]. 

## 5. Acute Treatment Options

If possible, a conservative approach to the acute management of SCAD is preferred and revascularization is reserved only for cases with high risk anatomy (left main coronary artery or left main coronary artery equivalent dissection) or clinical instability at presentation [1]. This is due to the high rate of technical failure of percutaneous coronary intervention and lack of protective effect against future SCAD events when PCI is performed [24], as well as the fact that in follow-up, most dissections heal spontaneously [22]. Acute risks involved with PCI in the SCAD cohort include unable to successfully gain access to true lumen, propagating the dissection with repeated dye injections, guide catheter dissection and risk of PCI extending the dissection both distally and proximally potentially jeopardizing a large area of myocardium. The need for emergent bypass surgery has been reported in up to 13% of cases where PCI was attempted [24].

Long-term risks of poor outcomes in PCI largely revolve around stent sizing and malapposition. In the acute setting, the stent may appear to be well-apposed to the wall but in time as the dissection heals and the hemorrhage resorbs, the stent may well be significantly undersized and malapposed, which predisposes the patient towards repeat revascularization and other catastrophic outcomes like stent thrombosis and its attendant complications. Strategies for successful percutaneous intervention in SCAD patients have been described and include interventional wires selection strategies [25], use of OCT imaging identify and seal the dissection flap with spot stenting [26], a multistent strategy with IVUS guidance to place stents proximal and distal to the lesion to prevent medial hemorrhage propagation [27]. Detailed technical recommendations of PCI techniques to mitigate risk of failure are beyond the scope of this review.

Coronary artery bypass grafting is a reasonable initial revascularization option if necessary, however, likely due to the fact that most dissections heal, bypass grafts have a high rate of failure at three years follow-up [24].

## 6. Medical Management

Patients that undergo a conservative treatment strategy should be observed in the hospital for 3 to 5 days as extension of the intramural hemorrhage can cause compression of the true lumen necessitating revascularization. Anticoagulation in the acute setting must weigh risk benefit of reduction in thrombus burden versus extension of the intramural hemorrhage. Outside of alternative indications for anticoagulation once the diagnosis of SCAD is made anticoagulation is recommended to be stopped [1].

In a cohort of 327 patients followed prospectively, beta blockers appear to have a protective effect against SCAD recurrence [3] though it should be noted that the majority of patients were discharged on beta blockers and this was not a study designed or powered to specifically address the protective effect of beta blockers against recurrence. Further validation in larger cohorts is required.

Statins are not routinely recommended outside of preexisting indications [1]. SCAD is by definition nonatherosclerotic and despite the putative endothelial effects of statins there is no evidence base for statins providing benefit in the SCAD population. A small single center retrospective study showed that statin use was associated with higher risks of SCAD recurrence [28]. This finding has not been replicated in larger series, nevertheless without proof of benefit statins should not be given routinely.

Antiplatelet regimens in SCAD remains an area of intense research and in need of further study. There is concern that given the underlying pathophysiology of medial hemorrhage that antiplatelet therapy could cause harm; simultaneously, however, there is concern regarding the risk of thrombus formation secondary to the intimal dissection. It is not well-established which treatment regimen balances the risk of medial hemorrhage extension versus prevention of thrombosis. An Italian series reported the results of 134 SCAD patients treated with both dual antiplatelet therapy (DAPT) and single antiplatelet agents. Bleeding outcomes were not reported but long term outcomes did not differ significantly between groups. As this was not a randomized trial, inherent differences between patients chosen for DAPT versus single antiplatelet strategy may confound results [29]. Some authors recommend the routine utilization of DAPT in the acute setting regardless of whether or not the patient received a stent with continuation of aspirin alone after one year [30], others reserve DAPT for those treated with percutaneous intervention and use aspirin for those managed conservatively [31]. Patients receiving stenting should be treated with DAPT in accordance with ACS guidelines [32].

Angiotensin converting enzyme inhibitors (ACE) and/or angiotensin receptor blockers (ARB) are useful in patients with residual left ventricular dysfunction though it should be kept in mind that this class of medications is teratogenic [1]. There is no evidence that use of decreased risk of SCAD recurrence with ACE/ARBs. However, the use of ACE/ARB after anterior STEMI is a Class I recommendation and a Class IIa recommendation for patients without heart failure or anterior STEMI. The recommendations for ACE/ARB are similarly strong for NSTEMI [33,34].

Chest pain post-SCAD is common among patients. The dissection itself often causes pain that needs to be distinguished from myocardial ischemia. Alternatively, patient may have residual small vessel dysfunction. Antianginal therapies such as nitrates, calcium channel blockers or ranolazine can be utilized in an effort to reduce symptoms.

## 7. Follow Up

Unfortunately, the risk of SCAD recurrence is high. At three years, the risk of recurrent de novo SCAD is around 10% [3]. Patients frequently re-present with chest pain that poses a significant diagnostic dilemma. Patients that have an established diagnosis of SCAD who are presenting with chest pain should be evaluated initially in according with standard chest pain protocols. If ischemia is objectively diagnosed patients should be taken back for coronary angiography to assess for high risk anatomy [1]. If patients are presenting with stable exertional symptoms, perfusion imaging or alternative modalities of stress testing are appropriate. Depending on the initial SCAD anatomy and the length of time that has elapsed from the initial SCAD diagnosis it may be reasonable to perform a coronary CTA to look for healing or worsening of already known dissections. The limitations of coronary CTA have been addressed above but in certain cases may be useful.

Patients diagnosed with SCAD should have vascular imaging from brain to pelvis in order to detect extracoronary vascular abnormalities as SCAD is highly associated with fibromuscular dysplasia and intracranial aneurysms [11]. 

Post-SCAD patients should be encouraged to avoid valsalva during exercise as well as extremes in temperature. Vancouver General Hospital has published a description of a SCAD cardiac rehabilitation program that focuses on mostly aerobic exercises with generous warm-up and cool-down periods, high repetitions of light weights and avoidance of lifting greater than 20 pounds [35].

## 8. Genetics

Genetic testing of patients diagnosed with SCAD without FMD is being offered at Massachusetts General Hospital, where a panel of molecular genetic tests have identified a pathologic gene mutation in COL3A1, a causative gene in vascular Ehler–Danlos as present in 4.1% of the SCAD population at their tertiary referral center [36]. Other genes have been identified including the F11R gene which encodes for the F11 receptor, a junctional adhesion molecule A, that concerns the regulation of tight junction assembly in endothelial cells [37]. However, at this time, genetic testing is of questionable clinical benefit and is not routinely recommended. Pathogenic mutations are infrequently identified and often mutations of unknown significance raise more questions with the possibility for further downstream testing that is of questionable clinical benefit [38]. Genetic testing at this juncture should be reserved for research purposes at appropriate referrals centers to gain more insight into this disease process. 

## 9. Gaps in Knowledge

Due to the relatively rare nature of this disease, randomized control trials are lacking to help guide therapy and develop risk predictor models with respect to incidence and recurrence. The SCAD Alliance has funded the iSCAD registry that seeks to serve as a repository of data for SCAD research as well as to link patients into a community. Hopefully, as awareness of this disease increases, more patients will be enrolled in registries that will shed light on providing effective, evidence-based therapies.

## Figures and Tables

**Table 1:** Saw Classification.

Type	Angiographic Characteristics
Type 1	Multiple lumen and contrast staining of the arterial wall
Type 2a	Stenosis of the artery with abrupt change in vessel caliber, typically >20 mm, not involving the angiographic tip of the vessel
Type 2b	Stenosis of the artery with abrupt change in caliber, extending to the angiographic tip of the vessel
Type 3	Mimics atherosclerosis

**Table 2:** Lists-associated conditions and suggested actions for patients presenting with SCAD.

Risk Factor	Suggested Action
Fibromuscular dysplasia	Brain to pelvis vascular imaging
Connective tissue disorder	No screening suggested
Systemic inflammatory disorders	No screening suggested
Pregnancy	Future pregnancies recommended against
Hormonal therapy	Discontinue
Intense valsalva	Avoid intense exercise
Intracranial aneurysm	Brain to pelvis vascular Imaging
